# 1,25 (OH)_2_D_3_ treatment alters the granulomatous response in *M. tuberculosis* infected mice

**DOI:** 10.1038/srep34469

**Published:** 2016-10-04

**Authors:** Kamlesh Bhatt, Wasiulla Rafi, Neel Shah, Sylvia Christakos, Padmini Salgame

**Affiliations:** 1 Department of Medicine, Center for Emerging Pathogens, Rutgers, New Jersey Medical School, Newark, NJ, USA; 2 Department of Microbiology, Biochemistry and Molecular Genetics, Rutgers, New Jersey Medical School, Newark, New Jersey, USA

## Abstract

Induction of cathelicidin-mediated antimicrobial pathway against intracellular *M. tuberculosis* by 1,25-dihydroxyvitamin D_3_ (1,25(OH)_2_D_3_), the active form of vitamin D, has been documented *in vitro*. However, in *in vivo* studies related to inflammatory disorders, 1,25(OH)_2_D_3_ has been demonstrated to induce an anti-inflammatory response. We therefore examined whether in the murine model of tuberculosis, the anti-inflammatory effects of 1,25(OH)_2_D_3_ would affect the outcome of *M. tuberculosis* infection. We show here that administration of 1,25(OH)_2_D_3_ to *M. tuberculosis* infected mice led to a change in lung granuloma architecture, characterized by a marked decrease in B cell lymphocytic aggregates. Consistent with the altered granulomas, 1,25(OH)_2_D_3_-treated mice also exhibited significantly higher bacterial burden in the lungs compared to the control group. These findings highlight the need to further investigate the effect of vitamin D on host immunity to *M. tuberculosis* in the context of the granulomatous response.

Vitamin D exerts regulatory control over a multitude of biological functions, including immune regulation^1^,^2^. The synthesis of the physiological form of vitamin D, vitamin D_3_ (cholecalciferol) is initiated with the photolysis of 7-dehydrocholestrol in the skin. Upon UVB radiation exposure, 7-dehydrocholestrol is converted to pre-vitamin D_3_ which is subsequently isomerized to vitamin D_3_. Conversion of vitamin D_3_ to 25-hydroxyvitamin D_3_ (25(OH)D_3_) takes place in the liver, and subsequently to 1,25-dihydroxyvitamin D_3_ (1,25(OH)_2_D_3_) in the kidney. Extra-renal conversion of 25(OH)D_3_ to 1,25(OH)_2_D_3_ occurs in other tissue types including immune cells such as dendritic cells, macrophages and T cells^3^,^4^,^5^. 1,25(OH)_2_D_3,_ the bioactive form of vitamin D mediates its effect in various cell types by binding to its nuclear receptor known as vitamin D receptor (VDR) which functions as a heterodimer with the retinoid X receptor and together with co-regulatory complexes, affects the transcription of target genes^5^,^6^,^7^,^8^.

Previous studies have noted that 1,25(OH)_2_D_3_ restricts intracellular *Mycobacterium tuberculosis* (Mtb) replication via the induction of anti-microbial peptides^9^. The convergence of Interleukin (IL)-1β and VDR signaling pathways in antimicrobial response has also been reported^10^,^11^. The vitamin D-mediated anti-microbial pathway can be activated by CD40L and IFNγ^12^. 1,25(OH)_2_D_3_ also inhibits intracellular growth of Mtb by interfering with the accumulation of infection-induced lipid droplets^13^. In contrast to its antimicrobial host protective functions, 1,25(OH)_2_D_3_ also down-modulates proinflammatory adaptive immune responses^14^,^15^,^16^,^17^. 1,25(OH)_2_D_3_ inhibits differentiation of dendritic cells resulting in the suppression of the proinflammatory cytokine IL-12 and an increase in the anti-inflammatory cytokine IL-10^18^,^19^. Similarly, a number of *in vitro* studies suggest that 1,25(OH)_2_D_3_ induces differentiation of T regulatory cells^20^,^21^,^22^. In murine models of autoimmune diseases, 1,25(OH)_2_D_3_ has been reported to suppress the generation of Th1 and Th17 effector cells^23^,^24^,^25^,^26^,^27^; the same T cell subsets that are involved in protection against tuberculosis^28^,^29^. With the identification of many immunomodulatory properties of 1,25(OH)_2_D_3_, interest in vitamin D supplementation as a therapeutic approach to treat chronic inflammatory diseases is gaining momentum. Although a definite link between vitamin D supplementation and amelioration of disease severity is yet to be established, several studies show an improvement in clinical outcome. Treatment of patients with remitting multiple sclerosis (MS) with a 6 month supplementation of high dose dietary vitamin D_3_ resulted in beneficial immunomodulatory effects^30^. In another randomized placebo controlled trial, high dose vitamin D supplementation led to decreased inflammatory cytokine levels and moderate clinical improvement in patients with systemic lupus erythematosus (SLE)^31^. A randomized, double-blind, placebo-controlled trial in adults with chronic obstructive pulmonary disease (COPD) indicated that vitamin D3 supplementation reduced the risk of severe exacerbations^32^. Because 1,25(OH)_2_D_3_ can initiate potent anti-inflammatory response in the host and suppress Th1 and Th17 responses^26^,^27^,^33^, it becomes imperative to ascertain whether 1,25(OH)_2_D_3_ treatment will have any repercussions on the induction of host protective response against Mtb infection.

Cathelicidin and β defensin genes, that have been shown to play a key role in anti-mycobacterial mechanisms in humans^34^, are not regulated by VDR in rodents due to the lack of vitamin D response elements (VDRE)^34^. However, a large number of VDR responsive lymphoid and myeloid cell functions have been studied in murine models of infection and autoimmune disease. Administration of 1,25(OH)_2_D_3_ was shown to protect against experimental autoimmune encephalomyelitis (EAE) as well as against experimental inflammatory bowel disease (IBD)^25^. Vitamin D was also reported to suppress proinflammatory immune response in experimental cerebral malaria in mice^35^. 1,25(OH)_2_D_3_ treatment resulted in increased susceptibility to *C. rodentium* infection^27^ and its infusion in *M. paratuberculosis*-infected mice led to exacerbation of the disease resulting in increased bacterial burden^36^. Overall these studies suggest that the murine model can be used to investigate the impact of vitamin D on host resistance against Mtb, which has remained undetermined.

In this study, we therefore, investigated whether administration of 1,25(OH)_2_D_3_, the active form of vitamin D, would affect host immunity to Mtb in a murine model of tuberculosis. We report here that Mtb-infected mice treated with 1,25(OH)_2_D_3_ exhibited altered pulmonary granuloma formation and reduced ability to contain bacterial burden in the lung as compared to the control group of mice. These findings are significant since they provide a framework to further explore the potential role of vitamin D in disrupting the inflammatory networks involved in granuloma formation and control of Mtb growth.

## Results

### Administration of 1,25(OH)_2_D_3_ during Mtb infection alters cellular recruitment to the lungs

**Figure 1:**
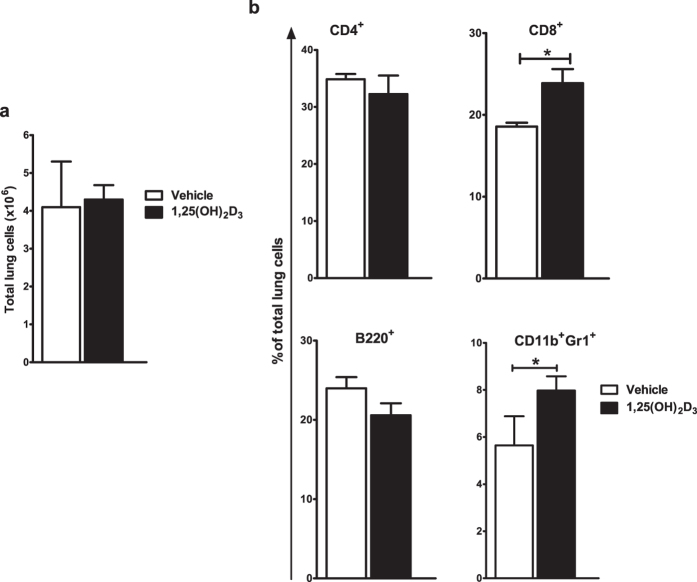
1,25(OH)_2_D_3_ treatment leads to altered cellular recruitment to the lungs in Mtb infected mice. Lung single cell suspensions were prepared from 1,25(OH)_2_D_3_-treated and control groups of mice at four weeks post Mtb infection. Total lung cells were counted via Trypan blue dye exclusion method (**a**). Percentage of CD4^+^, CD8^+^, B220^+^ and CD11b^+^Gr1^+^ cells in the lungs were quantitated by flow cytometry (**b**). Five mice were used in each group and data are presented as mean ± SD.

In a low dose aerosol exposure model, Mtb infection leads to an increased recruitment of various immune cell types to lungs that reaches a peak at four weeks post infection^28^,^37^. Therefore, in order to investigate the impact of vitamin D on lung cellularity during this acute phase of Mtb-infection, 1,25(OH)_2_D_3_-treated and control mice were sacrificed at four weeks post infection and flow cytometric analysis of lung single cell suspensions was carried out. The total number of cells recruited to the lungs of Mtb infected mice was observed to be the same in both the groups (Fig. 1a). Characterization of these cell populations was carried out via FACS (Fig. S1). We observed similar recruitment of CD4^+^ T cells in the lungs of both the groups of mice, however, percentage of CD8^+^ T cells was observed to be increased in the lungs of 1,25(OH)_2_D_3_-treated mice (Fig. 1b). We also observed a lower percentage of B220^+^ B cells, albeit not statistically significant and a significant increase in the percentage of CD11b^+^Gr1^+^ neutrophils in the lungs of 1,25(OH)_2_D3-treated mice compared to the control group (Fig. 1b).

### 1,25(OH)_2_D_3_ administration alters the organization of pulmonary granulomas

**Figure 2:**
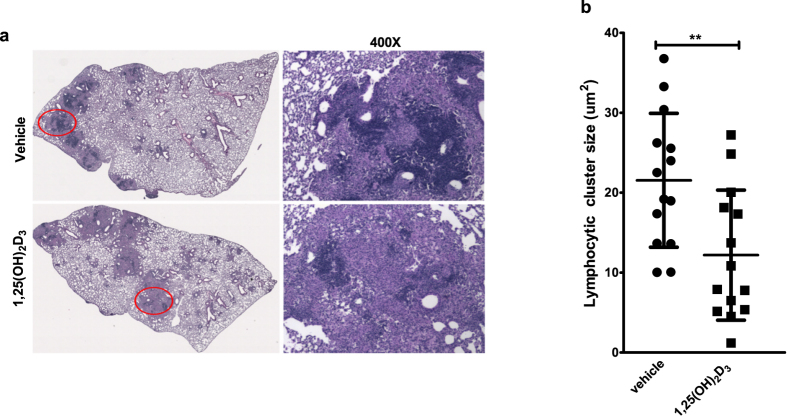
Lymphocytic cluster size is reduced in the pulmonary granulomas of 1,25(OH)_2_D_3_-treated mice. Formalin-fixed, paraffin-embedded lung tissue sections at six weeks post infection were stained with H&E (**a**). Nikon Microphoto FXA equipped with Objective Imagining surveyor and a motorized stage and NIS Elements Advanced Research software were used to calculate the lymphocytic cluster size in the granulomas of these mice. Each point in the graph represents surface area of one granuloma. Granulomas sampled from the left lung of five mice in each group are plotted (**b**) and are presented as mean ± SD. Lungs from all the animals in each group were used for analysis and representative image from each group is shown in Fig. 2a.

**Figure 3:**
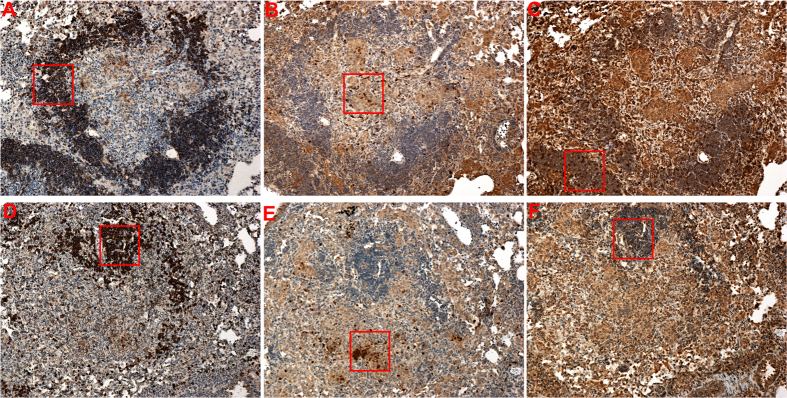
B cell rich lymphocytic cluster are diminished in the granulomas of 1,25(OH)_2_D_3_-treated mice. Immunohistochemistry was performed on lung tissue sections at six weeks post infection to detect CD20^+^ (**A, D**), Ly6G^+^ (**B, E**) and CD3^+^(**C, F**) cells in the lymphocytic clusters. Tissue sections were stained individually with antibodies against indicated surface markers. Image (100X) was captured using Nikon Eclipse E 800 microscope. The boxed area in each section is presented at higher magnification (200X) in Fig. S2. Lungs from all the animals in each group were used for analysis and representative images from each group are shown here.

In the murine model of tuberculosis, the acute phase of infection is followed by establishment of a granuloma that consists of small foci of lymphocytic aggregates interspersed with macrophages and other cell types^38^,^39^,^40^. The granulomatous response leads to containment of inflammation and controls bacterial growth^41^. Since at four weeks post infection lung single cell suspension derived from the two groups of mice exhibited unique cellular pattern, we therefore compared the subsequent development of inflammatory lesions in the lungs of 1,25(OH)_2_D_3_-treated mice to that of control mice at six weeks following Mtb infection. A similar level of inflammation was observed in both groups of mice (Fig. 2a). However, lymphocytic aggregates in the inflammatory zones were significantly smaller in size in the 1,25(OH)_2_D_3_-treated lungs compared to the control group (Fig. 2b). Immunohistochemical evaluation of lung sections showed that these lymphocytic clusters are rich in CD20 positive B cells (3A) and are associated with Ly6G (3B) positive neutrophils. The granulomas of 1,25(OH)_2_D_3_-treated mice exhibited an overall decrease in CD20 (3D) cell staining but showed enhanced Ly6G^+^ cells (Fig. 3E).

### Mice treated with 1,25(OH)_2_D_3_ exhibit reduced ability to contain bacterial burden during the chronic phase of Mtb infection

**Figure 4:**
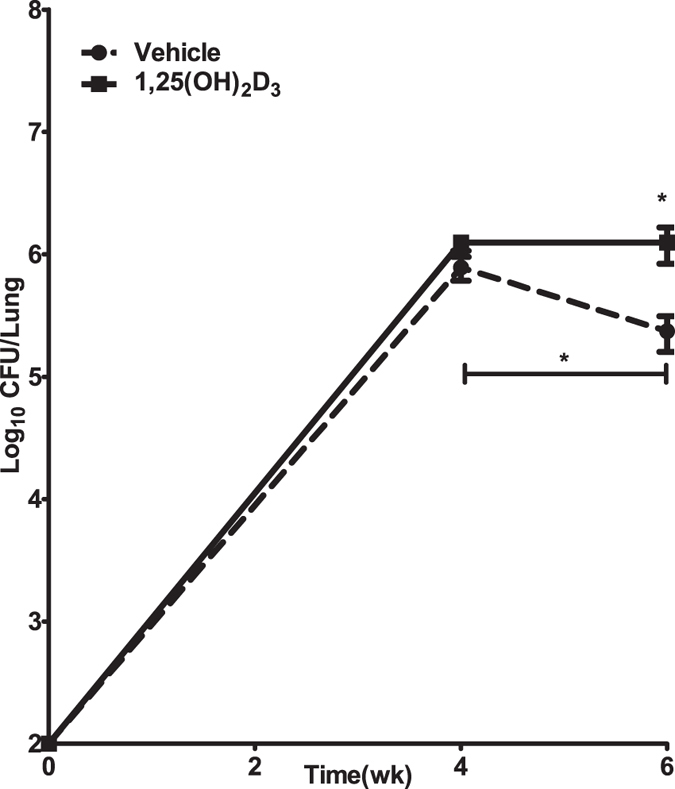
1,25(OH)_2_D_3_-treated mice fail to control the bacterial burden. C57BL/6 mice were treated with 1,25(OH)_2_D_3_ or vehicle and infected via aerosol with ~100 CFU of Mtb Erdman strain. Mice were sacrificed at four and six weeks following infection and viable bacterial burden was determined by plating the lung homogenates on 7H11 agar pates. Each time point includes five mice per group. Experiment was performed twice and data from one experiment is presented as mean CFU ± SD.

We next compared the bacterial burden in the two groups of mice at four and six week time intervals following Mtb infection. In the control group, as expected, there was an increase in bacterial burden at four weeks, and at six weeks as mice entered the chronic phase of infection, the bacterial burden decreased significantly (Fig. 4). In the 1,25(OH)_2_D_3_-treated mice, a similar increase in bacterial burden was also seen at four weeks, but, unlike in the control group, these mice did not exhibit a decrease in bacterial burden at six weeks and there was a significant difference in the bacterial burden in the two groups of mice at this stage (Fig. 4).

### 1,25(OH)_2_D_3_-treated mice exhibit an overall increase in inflammatory gene expression

**Figure 5:**
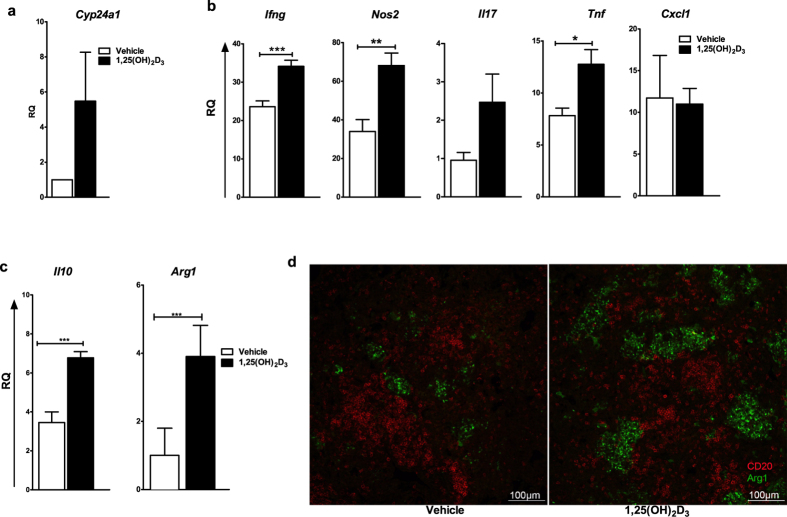
Enhanced expression of cytokines in the lungs of 1,25(OH)_2_D_3_-treated mice and *in situ* detection of arginase-1. Total RNA was isolated from the lungs of of Mtb infected mice at four weeks post infection. Gene expression was assessed by real-time RT-PCR and fold induction in gene expression compared to uninfected lungs was determined (**a, b, c**). Immunofluorescence was used to detect arginase-1 expression *in situ* (**d**). Lung sections from mice infected with Mtb at four weeks post infection were stained for CD20 (red) and arginase-1 (green). Lungs from all the animals in each group were used for analysis. Representative sections from each group are shown. For RT PCR, five mice were used in each group and data are presented as mean ± SD.

We next evaluated the gene expression profile of key inflammation-related genes including *Cyp24a*, at four weeks post infection by real-time PCR. We observed that *Cyp24a* expression was induced in 1,25(OH)_2_D_3_ treated group of animals (Fig. 5a). Increased expression of *Ifng*, *Nos2*, *Il17*, and *Tnf* was observed in 1,25(OH)_2_D_3_-treated-mice compared to control mice (Fig. 5b). The 1,25(OH)_2_D_3_-treated-mice also exhibited significantly elevated gene expression for *Il10* and *Arg1* (Fig. 5c), indicating that the overall increase in both pro- and anti-inflammatory genes is likely a reflection of the increased bacterial burden in the lungs of these mice. Immunofluorescence staining confirmed the increased expression of arginase -1 in the lungs of 1,25(OH)_2_D_3_-treated mice (Fig. 5d).

## Discussion

Host resistance to Mtb infection is critically dependent on the complex interplay between innate and adaptive immune responses to the pathogen^42^. Although proinflammatory responses are key to the host’s ability to contain the infection, anti-inflammatory immune response pathways are critical for the prevention of excessive inflammation-induced damage to the host^42^. Vitamin D deficiency and VDR polymorphism are associated with increased susceptibility or progression to tuberculosis disease^43^,^44^,^45^. It has also been suggested that vitamin D as an adjunctive therapy during tuberculosis treatment may accelerate clinical recovery and inflammation resolution^46^,^47^. Although a number of studies conducted *in vitro* strongly suggest that 1,25(OH)_2_D_3_ directly and indirectly activates host antimicrobial pathways^9^,^11^,^48^, there is a lack of *in vivo* studies that focus on immunomodulatory role of vitamin D in the context of Mtb infection. The key innate and adaptive immune response pathways generated in response to Mtb infection bear similarities in humans and mice. The availability of mice strains that exhibit disease pathology similar to humans is increasingly making this model an attractive tool in preclinical testing of drugs and vaccines^49^,^50^,^51^. Therefore, in this study we sought to employ the murine model of tuberculosis to investigate the impact of 1,25(OH)_2_D_3_ on the outcome of Mtb infection. Our data indicating altered cellular recruitment to the lungs and change in granuloma architecture of 1,25(OH)_2_D_3_-treated mice, suggest an important role for vitamin D in regulating Mtb infection induced immune response. Particularly, the reduction in the size of B cell rich lymphocytic clusters in the 1,25(OH)_2_D_3_ -treated lungs suggest that vitamin D may affect the immune mechanisms involved in B cell follicular response in the tuberculosis granuloma with consequent impact on the host’s ability to control Mtb growth.

Several studies have indicated that B cell functions are modulated by 1,25(OH)_2_D_3_^6^,^52^,^53^,^54^,^55^,^56^. It has been reported that although B cells do not express VDR constitutively^52^,^57^, VDR expression is induced by B cell activating signals^52^,^55^. Whether 1,25(OH)_2_D_3_ directly regulates B cells has been a matter of debate. It has been suggested that 1,25(OH)_2_D_3_ mediated inhibition of B cell function may be indirect, through the modulation of T cell or monocyte functions^53^,^58^. B cell rich lymphocytic aggregates, bearing features of secondary lymphoid follicles, are characteristic of both human and murine tuberculosis granulomas^38^,^39^,^59^,^60^. B cell follicles in tuberculosis granuloma have been suggested to provide a site for continuing cellular proliferation in response to Mtb antigens and have been implicated in the regulation of the granulomatous response^61^. In a study by Chen *et al.*^6^, 1,25(OH)_2_D_3_ was shown to inhibit the proliferation of activated B cells and induce their apoptosis *in vitro.* However, we were not able to detect enhanced apoptosis in the lymphocytic aggregates in the 1,25(OH)_2_D_3_-treated mice compared to the control group. Mtb infection in B cell deficient mice^38^ resulted in an increased influx of neutrophils to the lungs, higher expression of IL-17^62^ and increased bacterial burden. It is thus plausible that modulation of B cell responses by 1,25(OH)_2_D_3_, either directly or indirectly, may be a contributing factor to the observed increase in the number of neutrophils in the lungs of 1,25(OH)_2_D_3_-treated mice. Because of the altered granuloma environment, 1,25(OH)_2_D_3_-treated mice are subsequently unable to restrict bacterial burden in the lungs as efficiently as control animals as they enter the chronic phase of infection. Although we observed increased expression of several pro-inflammatory cytokines, expression of IL-10 and arginase-1 was also increased in 1,25(OH)_2_D_3_-treated mice. Macrophage-derived IL-10 induces arginase-1 in alternatively activated macrophages^63^, and furthermore, arginase-1 controls Mtb growth and T cell mediated immunopathology^64^. Therefore, future studies should evaluate how these cytokines modulate the inflammatory response in acute infection and also the immunopathology that is induced in chronic infection in 1,25(OH)_2_D_3_-treated mice.

In a recent study, Reeme *et al.*^65^ reported that high dietary vitamin D suppressed proinflammatory cytokine response, accompanied by mitigated pulmonary immunopathology in late stage Mtb infection in C3HeB/FeJ mice. It is of interest that similar to this study^65^ we also observed increased proinflammatory cytokine response and neutrophil influx in 1,25(OH)_2_D_3_-treated mice. Although, in the Reeme study, the authors did not characterize B cells in the lymphocytic clusters, they observed a reduction in lymphocytic cluster size in the groups of mice that were fed high vitamin D diet. The reduction in B cell rich lymphocytic cluster size in response to 1,25(OH)_2_D_3_ treatment suggests that vitamin D may modulate B cell mediated immune response to Mtb infection. Our future experiments will dissect the mechanisms involved in vitamin D mediated modulation of B cell function in Mtb infection. Another difference between our finding in the C57BL/6 mice and the C3HeB/FeJ data is that the latter study did not find increased bacterial numbers in the lung of mice fed high dietary vitamin D. The increased bacterial burden in response to 1,25(OH)_2_D_3_ seen in our study may be the result of mouse genotype specific differences. It is also possible that the strain of bacteria may influence the outcome of vitamin D or 1,25(OH)_2_D_3_ treatment since our study used Mtb Erdman while the C3HeB/FeJ mice were infected with Mtb H37Rv. It is therefore critical that investigations are conducted on mice with varying genetic backgrounds such as the Diversity Outbred mice^66^ and with diverse clinical Mtb strains to delineate the effects of vitamin D on host immunity to Mtb. Another limitation of our study is that mice do not express human cathelcidin, which has been shown to be involved in antimicrobial activity against Mtb *in vitro*^9^. Further *in vivo* studies are needed in LL37 transgenic mice (expressing the human cathelicidin gene) to fully evaluate the interplay between vitamin D and the host immune response during Mtb infection and to dissect the underlying mechanisms.

## Methods

### Mice

C57BL/6 female mice (6–8 weeks old) were purchased from the National Cancer Institute (Frederick, MD, USA). Mtb-infected mice were housed in the animal BSL3 facility and guidelines from R-NJMS-Institutional Animal Care and Use Committee were followed in handling the animals. All experimental protocols in this study were approved by R-NJMS-Institutional Animal Care and Use Committee.

### Determination of pulmonary bacterial burden

1,25(OH)_2_D_3_ was obtained from Cayman Chemical Company, (Ann Arbor, MI, USA). Mice were injected with 20 ng 1,25(OH)_2_D_3_ or vehicle (90% 1,2-propanediol in ethanol) via subcutaneous route three times a week. The dose of 1,25(OH)_2_D_3_ was chosen on the basis of previous *in vivo* studies with mice^67^. The treatment was initiated a day before the infection and continued until the completion of the study. Mice were infected with a low dose of Mtb Erdman strain (Trudeau Institute, Saranac, NY) in a whole body inhalation exposure system (Glas-Col, LLC, Terre Haute, IN). The number of bacteria deposited in the lungs were determined by plating the whole lung homogenates on 7H11 plates 24 hrs following aerosolization. At each time interval studied, infected animals were sacrificed by cervical dislocation and the right superior lobe of the lung was homogenized in PBS containing 0.05% Tween 80. Serial dilutions of the homogenates were plated onto 7H11 agar. The plates were incubated at 37 °C and colonies counted after 21 days. The rest of the lung was reserved for single cell preparation, RNA extraction and histological studies.

### Lung single cell preparation

Lungs were perfused with 5 ml sterile PBS, cut into small pieces and incubated with 2 mg/ml collagenase D (Roche) for 30 min. The digestion was stopped by adding 5 mM EDTA. The digested tissue was transferred to a 40-μm nylon cell strainer and disrupted using a syringe plunger to obtain single cell suspensions. RBCs were lysed with ACK lysing buffer and viable cell number was determined by trypan blue dye exclusion method.

### Immunohistochemistry

Mtb-infected mice were sacrificed at indicated time intervals and lungs were perfused with PBS. Excised lungs were fixed in 4% paraformaldehyde for a week and subsequently stored in 70% ethanol until they were embedded in paraffin. Five micrometer sections were cut and stained using the standard H&E protocol. For immunohistochemistry, four to six micrometer sections were cut and mounted onto Superfrost/Plus microscope slides (Fisherbrand). Tissue sections were de-paraffinized with xylene and rehydrated with ethanol gradations and water. CD3, Ly6G, CD20 and arginase-1 epitopes were retrieved using heat induced epitope retrieval method as described previously^68^. The tissue sections were dipped in 10 mM citrate retrieval buffer (pH 6.0) and heated in a microwave pressure cooker (Nordic ware). Tissue sections were then blocked with Background Buster (Innovex Biosciences) for 30 minutes. Primary antibodies against CD20 (M-20), and arginase-1 (H-52), were obtained from Santa Cruz Biotechnology. CD3 (Rabbit polyclonal) was obtained from Abcam and Ly6G (1A8) was obtained from Biolegend. Sections were stained with these antibodies at 4 °C overnight. The sections were washed (PBS containing 0.5% tween 20) and reacted with biotinylated (1:100, Vector Laboratories) or fluorescent-labeled secondary antibody (1:1000 Life Technologies) for 45 minutes at room temperature. For fluorescent detection of CD20 and arginase-1, Alexa 568 and Alexa 488 conjugated donkey – anti rabbit (Life Technologies) secondary antibodies were used. Relevant isotype controls were used for each primary antibody. The streptavidin horseradish peroxidase substrate (BioGenex) was used for immunodetection using DAB as a chromogen (BioGenex). The sections were counterstained with hematoxylin, and subsequently dehydrated in 95% and 100% ethanol followed by xylene. Finally, they were mounted on coverslips for microscopic visualization. Sections stained with fluorescent-labeled antibodies were directly mounted with ProLong antifade mounting media (Molecular Probes). Nikon Microphot-FXA, equipped with Objective Imaging Surveyor and a motorized microscope stage was used to capture tiled images. Granuloma area was measured using NIS Elements Advanced Research software. Fluorescent images were captured using NikonA1R laser scanning confocal microscope equipped with 20X planApo - numerical aperture 0.75.

### Flow cytometry

The following anti-mouse mAbs used for the study: anti-CD4 (RM4–5), anti-CD8 (Ly-2), anti-B220 (RA3-6B2), anti-CD11b (M1/70) and anti-Ly-6G & Ly-6C (RB6-8C5) were purchased from BD Biosciences. All antibodies were directly conjugated to fluorochromes and isotype controls were included for each antibody type. Single cell suspensions from the lungs were made as described above and cell density was determined. Approximately one million cells were washed and re-suspended in FACS buffer containing appropriate concentrations of fluorochrome-conjugated antibodies. After thirty minutes incubation at 4°C, the cells were washed again in FACS buffer and fixed with 4% paraformaldehyde for 30 min. The cells were acquired on BD LSR II flow cytometer and data were analyzed using FlowJo (Tree Star).

### Real-Time RT PCR

Lung tissues were homogenized in TRIzol^®^ (Ambion™) and stored at −80 °C till further processing. RNA was extracted from TRIzol^®^ and further purified using RNeasy kit (Qiagen) and reverse transcribed using High Capacity RNA to cDNA kit (Applied Biosystems™). cDNA was amplified using Taqman^®^ reagents (Applied Biosystems™) on the ABI PRISM 7900 HT Sequence Detection System and fold induction in gene expression relative to uninfected tissue (RQ) was calculated by 2^−ΔΔCT^ method^69^ by the Applied Biosystems software. Briefly, relative gene expression (fold induction) is calculated as 2^−ΔΔCt^, where ΔCt = Ct (gene of interest) – Ct (normalizer = β-actin) and the ΔΔCt = ΔCt (sample) – ΔCt (calibrator). The calibrator in our study is lung tissue obtained from uninfected mice. Due to the undetectable *Cyp24a1* amplification in uninfected tissues, a Ct value of 40 was assigned to calculate relative fold induction. The following primer/probe sets from Applied Biosystems™ were used; *Nos2* (Mm00440502_m1), *Tnf* (Mm00443260_g1), *Ifng* (Mm01168134_m1), *Il17a* (Mm00439618_m1), *Il10* (Mm00439614_m1), *Arg1* (Mm00475988_m1), *Cyp24a1* (Mm00487244_m), *Cxcl1* (Mm04207460_m1), and *β actin* (Mm00607939_s1).

### Statistical analysis

For statistical analysis, GraphPad Prism Software (version 5) was used. The unpaired Student *t* test was used to determine statistical significance between the two groups. Values of *p ≤ 0.05, **p ≤ 0.01 and ***p ≤ 0.001 were considered significant.

## Additional Information

**How to cite this article**: Bhatt, K. *et al.* 1,25 (OH)_2_D_3_ treatment alters the granulomatous response in *M. tuberculosis* infected mice. *Sci. Rep.* **6**, 34469; doi: 10.1038/srep34469 (2016).

## Supplementary Material

Supplementary Information
