# Supplementary material for: 1,25 (OH)2D3 treatment alters the granulomatous response in M. tuberculosis infected mice
Source: Sci Rep. 2016 Oct 4;6:34469. doi: 10.1038/srep34469 (PMC5048147; doi:10.1038/srep34469)
Supplement: Supplementary Information [file srep34469-s1.pdf]

1,25 $(OH)_2D_3$ treatment alters the granulomatous response in *M. tuberculosis* infected mice

Kamlesh Bhatt[1,*], Wasiulla Rafi[1,§], Neel Shah[1,‡], Sylvia Christakos[2] and Padmini Salgame[1]

[1] Department of Medicine, Center for Emerging Pathogens, Rutgers, New Jersey Medical School, Newark, NJ, USA.

[2] Department of Microbiology, Biochemistry and Molecular Genetics, Rutgers, New Jersey Medical School, Newark, New Jersey, USA.

[§] *Current address*: Ogilvy Commonhealth Worldwide, 440 Interpace Parkway, Parsippany, New Jersey, USA

[‡] *Current address*: Rowan University School of Osteopathic Medicine, Stratford, New Jersey, USA.

*****Correspondence to: Bhattka@njms.rutgers.edu

Supplementary Figures

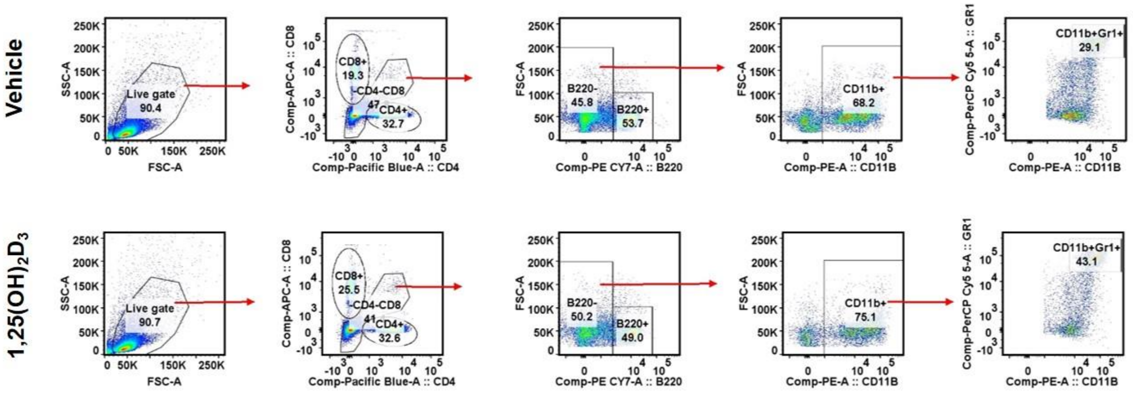

Figure S1: Sequential gating strategy to identify various cell populations (Figure 1) derived from single cell suspensions from the Mtb infected lungs.

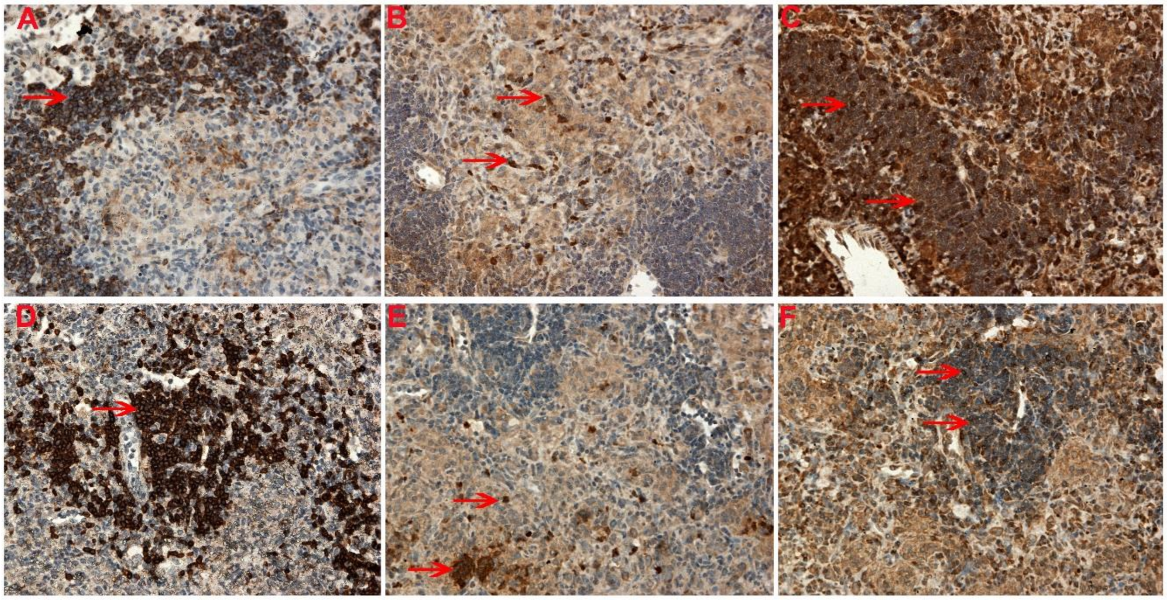

Figure S2: Boxed area in Figure 3 at 200X magnification. Arrows indicate the stained cells in the tissue.
